# A Cross-Sectional Study of Cardiovascular Autonomic Reactivity in Ehlers-Danlos Syndrome

**DOI:** 10.7759/cureus.64542

**Published:** 2024-07-14

**Authors:** Waqas Alauddin, Shahnawaz Alam, Mohit Mishra, Prajakta M Radke, Rishika Shree, Brishabh R Prajesh, Aparna Chaturvedi, Anant Patil, Tarun Kumar Singh, Malik Faizan Ahmad

**Affiliations:** 1 Physiology, Naraina Medical College and Research Centre, Kanpur, IND; 2 Physiology, MGM Medical College, Navi Mumbai, IND; 3 Medicine, Naraina Medical College and Research Centre, Kanpur, IND; 4 Pharmacology, D. Y. Patil University School of Medicine, Navi Mumbai, IND; 5 Forensic Medicine, Naraina Medical College and Research Centre, Kanpur, IND; 6 Community Medicine, Naraina Medical College and Research Centre, Kanpur, IND

**Keywords:** parasympathetic reactivity, sympathetic reactivity, autonomic dysfunction, cardiovascular reflex tests, ehlers-danlos syndrome

## Abstract

Background: Ehlers-Danlos syndrome (EDS) may be linked to dysfunction in the autonomic nervous system, affecting collagen production and processing. These collagen abnormalities lead to a condition called dysautonomia. Findings underscore the need for further investigation into autonomic nervous system function in EDS which involves larger studies to strengthen the evidence of non-invasive screening tools like cardiovascular reflex tests. These tests might offer a valuable way to assess an individual's risk for future complications.

Objective: This study aimed to assess autonomic reactivity in EDS patients using cardiovascular reflex tests.

Methods: This study was conducted at the Department of Physiology, Naraina Medical College and Research Centre, Kanpur (Uttar Pradesh). The cardiovascular reflex test was used to identify 60 individuals, 30 of whom were EDS patients and 30 were healthy controls, with a common age limit of 18-25 years. Cardiovascular reflex tests, such as the delta heart rate, lying-to-standing test (LST), deep breathing test (DBT), and Valsalva maneuver, were performed and recorded for each subject. IBM SPSS Statistics for Windows, Version 21.0 (Released 2012; IBM Corp., Armonk, New York, United States) was used for the compilation and analysis of data.

Results: The expiration-to-inspiration (E:I) ratio and delta heart rate of the EDS patients both significantly decreased as compared to the healthy control group (1.10±0.02 versus 1.22±0.77 and 14.03±0.31 versus 15.52±0.32). The Valsalva ratio of the EDS patients decreased (1.28±0.01) as compared to the healthy control group (1.46±0.01), which was statistically significant. The 30:15 ratio in the EDS patients was significantly decreased compared to the healthy control group (1.08±0.01 versus 1.15±0.01). The handgrip test and cold pressor test results were statistically insignificant.

Conclusion: The study suggests a connection between EDS and autonomic nervous system dysfunction, causing symptoms like tachycardia and hypotension. It recommends exploring non-invasive cardiovascular reflex tests as a tool to identify autonomic dysfunction in EDS patients and predict long-term cardiovascular complications. These tests offer valuable insights into autonomic function.

## Introduction

Ehlers-Danlos syndrome (EDS) is strongly associated with the autonomic nervous system, which is caused by affecting the production and processing of collagen which can be often referred to as dysautonomia [1]. Even after impacting at least one in 5000 individuals globally, EDS remains a group of underdiagnosed and often misunderstood heritable connective tissue disorders [2]. Multiple research studies have identified tachycardia and hypotension as established complications, potentially leading to the development of one of the four characteristic symptoms: postural tachycardia syndrome (POTS), neurally mediated hypotension (NMH) also referred to as vasovagal syncope or neurocardiogenic syncope, orthostatic hypotension (OH) or delayed orthostatic hypotension, and orthostatic intolerance (OI) [1,3,4]. These impaired cardiovascular parameters can significantly impact the quality of life of individuals with EDS limiting their daily activity and causing physical discomfort [3]. Autonomic dysfunction can eventually cause cardiac vascular morbidity and mortality; hence, this should be diagnosed as soon as possible [3,4]. As per previous studies, an increase in sympathetic activity and a lower parasympathetic tone were observed by using heart rate variability (HRV), thus causing autonomic dysfunction [4,5].

This autonomic resting tone can be evaluated by a cardiovascular reflex test. To the best of our knowledge, this will be the first study in which a cardiovascular reflex test will be performed in patients with EDS to assess the gap and address the lacunae between sympathetic and parasympathetic reactivity/tone.

## Materials and methods

This cross-sectional study was conducted in the Department of Physiology, Naraina Medical College and Research Centre in Kanpur, India, between January 2024 and May 2024. Following informed written consent, participants diagnosed with EDS, hypermobility type, were recruited from the medicine outpatient department (OPD). The study included two groups of 30 participants each, both sexes, age-matched between 18 and 25 years old. One group consisted of individuals diagnosed with EDS. The other group comprised healthy controls. To ensure a homogenous study population, individuals with pre-existing conditions such as autonomic dysfunction, hepatorenal or endocrine disorders, hypertension, diabetes, heart failure, neurological or psychiatric conditions, substance use disorders, or other significant medical conditions were excluded.

Study design includes methods for assessing the results of cardiovascular autonomic reactivity [6,7]. The research complied with established procedures by integrating a series of techniques to assess cardiovascular reflexes. These techniques comprised deep breathing maneuvers, transition from lying down to standing up (lying-to-standing test (LST)), the Valsalva maneuver, cold pressor test, and handgrip test. To minimize potential confusing impacts, participants were directed to refrain from consuming food or caffeine for four hours prior to the commencement of the experiment. Simultaneous monitoring of brachial artery blood pressure and lead II electrocardiogram (ECG) was carried out throughout the assessment procedures.

Deep breathing test (DBT)

The deep breathing assessment functions as an indirect evaluation of cardiac parasympathetic activity. This is due to the fact that deep breathing stimulates the vagus nerve, which has a crucial role in controlling HR. Therefore, this assessment is frequently known as a cardiovagal maneuver. Participants were instructed to execute slow, deep breaths, with each inhalation and exhalation lasting approximately 10 seconds. The deep breathing maneuver consisted of six cycles of slow, deep breaths, each lasting 10 seconds. Throughout this maneuver, ECG data was thoroughly examined to compute HR and the interval between heartbeats (RR interval) during both inhalation (inspiratory) and exhalation (expiratory) phases for each cycle.

The delta HR illustrates the extent of variation in HR between inhalation (highest HR) and exhalation (lowest HR) averaged across the six breathing cycles.

The expiration-to-inspiration (E:I) ratio measures the average proportional duration between the longest heartbeat interval during exhalation and the shortest interval during inhalation across the six deep breathing cycles.

Valsalva maneuver

The Valsalva maneuver is a valuable technique for assessing cardiovagal activity, which is an alternative expression for parasympathetic nervous system function. This maneuver evaluates how the HR reacts to changes in pressure caused by straining against a closed airway. Participants were instructed to forcefully exhale into a mouthpiece connected to a sphygmomanometer for 15 seconds, aiming to maintain an expiratory pressure of 40 mmHg. To focus on the effects of the Valsalva maneuver, deep breaths were abstained from immediately before and after the maneuver. The Valsalva ratio is obtained by dividing the RR interval with the longest duration (phase 4) by the RR interval with the shortest duration (phase 2).

Handgrip evaluation

The handgrip test evaluates sympathetic nervous system activity, specifically adrenergic function, by examining changes in diastolic blood pressure (DBP) in reaction to a controlled isometric handgrip exercise.

Cold pressor test

The cold pressor test assesses the activity of sympathetic adrenergic functions by observing the DBP reactions to cold stimuli. Initially, the baseline blood pressure is documented, following which the participant is directed to submerge their right hand in cold water (at 10°C) up to the wrist for a duration of one minute. Simultaneously, the blood pressure readings are taken from the arm opposite to the immersed hand. Any increase in DBP from the initial baseline level is recorded and analyzed.

Statistical analysis

This research utilized a licensed statistical software, IBM SPSS Statistics for Windows, Version 21.0 (Released 2012; IBM Corp., Armonk, New York, United States), to manage, analyze, and interpret the data. The researchers presented their findings using descriptive statistics, including means and standard deviations (SD) to summarize the data for each group (EDS individuals and control group). To assess if there were statistically significant differences between the two groups, they employed an unpaired t-test. Furthermore, the study examined relationships between multiple variables using Pearson's correlation coefficient. Additionally, regression analysis was likely used to explore how one variable might be predicted by changes in others. Throughout the analysis, a significance level of p<0.05 was maintained, indicating that only results with a less than 5% chance of occurring by random chance were considered statistically meaningful.

## Results

Sixty individuals with EDS and healthy individuals, ranging in age from 18 to 25, were included in this study. The mean HR of EDS patients was 99.83±2.94, whereas that of healthy individuals was 74.96±3.33 bpm (p=0.000*) as depicted in Table 1. The systolic blood pressure (SBP) of EDS patients was 105.50±3.29 mmHg, while that of healthy individuals was 118.07±2.59 mmHg (p=0.000*). The diastolic BP of the EDS patients was 64.37±2.74 mmHg, whereas that of the healthy individuals was 75.57±2.11 mmHg (p=0.000*), as shown in Table 1. Respiratory rate was significantly increased in EDS patients (20.10±0.64) as compared to the control group (17.00±0.68) (p=0.000*).

**Table 1:** Comparison of basal parameters between EDS individuals and control group Values are expressed as mean±SD or number (%). *p-value <0.05, statistically significant BMI: body mass index; HR: heart rate; SBP: systolic blood pressure; DBP: diastolic blood pressure; RR: respiratory rate; SD: standard deviation; NS: not significant; EDS: Ehlers-Danlos syndrome

	EDS (mean±SD)	Control (mean±SD)	P-value
Age (in years)	21±1.58	20.93±1.80	NS
BMI	21.33±1.12	21.50±1.04	NS
HR	99.83±2.94	74.96±3.33	0.000*
SBP	105.50±3.29	118.07±2.59	0.000*
DBP	64.37±2.74	75.57±2.11	0.000*
RR	20.10±0.64	17.00±0.68	0.000*

The delta HR (DBT) of EDS patients was 14.03±0.31, while that of healthy individuals was 15.52±0.32 (p=0.000*), as shown in Table 2. The E:I ratio (DBT) of EDS patients was 1.10±0.02, compared to 1.22±0.01 in healthy individuals (p=0.000*). Table 2 shows that the Valsalva ratio was 1.28±0.01 in EDS patients and 1.46±0.01 in healthy individuals (p=0.000*). The 30:15 ratio and fall in SBP (LST) in the EDS patients was significantly decreased (p=0.00*) as compared to the healthy control group. The rise in heart rate (LST) was significantly higher in EDS patients (30.42±3.14) as compared to the control group (10.49±3.07) (p=0.000*).

**Table 2:** Comparison of the cardiovascular reflex test between EDS patients and healthy control individuals The values are expressed as mean±SD. *p value <0.05, statistically significant Delta HR: delta heart rate; E:I: expiration-to-inspiration; Delta diastolic blood pressure: change in diastolic blood pressure in the isometric handgrip test; Delta systolic blood pressure (fall in SBP): systolic blood pressure during the lying-to-standing test; DBT: deep breathing test; LST: lying-to-standing test; SD: standard deviation; EDS: Ehlers-Danlos syndrome

	EDS (mean±SD)	Control (mean±SD)	P-value
Delta HR (DBT)	14.03±0.31	15.52±0.32	0.000*
E:I ratio (DBT)	1.10±0.02	1.22±0.01	0.000*
Valsalva ratio	1.28±0.01	1.46±0.01	0.000*
30:15 ratio (LST)	1.08±0.01	1.15±0.01	0.000*
Fall in SBP (LST)	-13.63±1.54	5.60±1.63	0.000*
Rise in heart rate (LST)	30.42±3.14	10.49±3.07	0.000*
Delta DBP-handgrip test	14.39±2.72	14.80±2.85	0.98
Cold pressor test	11.87±3.19	12.80±2.02	0.18

 The handgrip test and cold pressor test results were statistically insignificant.

## Discussion

A study revealed that individuals with EDS exhibited notably elevated baseline levels for crucial cardiovascular parameters such as resting HR, SBP, and DBP in comparison to a healthy cohort [4]. Our findings revealed increased basal cardiovascular metrics (resting HR, SBP, and DBP) detected in EDS patients which can be attributed to autonomic dysfunction. This dysfunction is believed to be triggered by an excessive production of catecholamines, which excessively activate alpha- and beta-adrenergic receptors [4].

Tests evaluating parasympathetic nervous system function (e.g., cardiovascular reflex test), including the DBT and its associated measurements (E:I ratio, delta HR, and Valsalva), displayed a significant reduction in EDS patients when contrasted with healthy controls. Prior studies have established a correlation between a notable decline in the Valsalva ratio and HR, indicating diminished parasympathetic activity and sympathoexcitation in these individuals. This phenomenon is predominantly caused by the heightened responsiveness of alpha- and beta-adrenergic receptors [4]. In our study, EDS demonstrated reduced parasympathetic tone as evidenced by lower findings on the parasympathetic reactivity tests (E:I ratio and delta HR values).

An assessment of the autonomic nervous system in EDS patients disclosed distinctive alterations in comparison to the control group. During the transition from lying to standing (a test of sympathetic function), a considerable reduction in SBP was observed. Furthermore, a pivotal indicator of parasympathetic function, the 30:15 ratio, exhibited a noteworthy decrease in EDS patients. Interestingly, these findings coincided with an escalation in HR. This hypothesis is substantiated by the following plausible explanations [2].

Baroreceptor dysfunction causing venous pooling in the lower extremities due to gravity prompts baroreceptors to regulate blood flow back to the body. However, due to connective tissue laxity, the blood vessels are unable to constrict, resulting in the release of catecholamines. This release leads to tachycardia and a decrease in SBP [5,8,9].

When transitioning from a supine to an upright position, the study noted a perplexing occurrence in EDS patients: a surge in HR (tachycardia) concomitant with a substantial decline in SBP. This seemingly contradictory response can be elucidated by the body's exaggerated reaction to changes in blood circulation [5,8,9]. Our findings support this conclusion.

EDS could instigate dysfunction in the autonomic nervous system, responsible for regulating involuntary functions such as HR and vascular constriction. This dysfunction might trigger the excessive release of stress hormones, namely, catecholamines like adrenaline and noradrenaline [5,9].

These catecholamines typically induce the body's "fight-or-flight" response, augmenting HR and vascular constriction. Nonetheless, in EDS patients, the adrenergic receptors (cell receptors responsive to catecholamines) might be hypersensitive [2]. This hyperresponsiveness could elucidate the elevation in HR despite a decline in blood pressure.

The possibility of finding cardiovascular diseases in EDS patients that are identified by mitral valve prolapse is associated with excessive catecholamines, orthostatic intolerance, and occasional dysrhythmias [10].

Another mechanism of orthostatic hypotension in patients of EDS is connective tissue laxity which can be due to the mutation in the COL1A1 gene, associated with EDS, that may result in defective collagen synthesis [11]. This weakens connective tissues throughout the body, potentially impacting venous function [11]. Weakened veins may struggle to efficiently propel blood, leading to pooling and disrupting overall hemodynamics [11]. Consequently, a decline in blood pressure ensues, culminating in hypotension and potentially inducing syncope [11]. Our findings also reveal the same.

EDS patients often experience abnormal heart function, and research suggests histamine, a molecule with diverse cardiovascular effects, might play a role [12]. Histamine can cause both a drop in blood pressure (hypotension) and an increased HR (tachycardia) [12]. Interestingly, studies have identified the activation of mast cells, which release histamine, in EDS patients. Additionally, histamine can trigger vasodilation, potentially contributing to hypotension [12]. Furthermore, it can indirectly lead to an excess release of catecholamines, hormones that can counteract the initial blood pressure drop and increase HR. This complex interplay between histamine and the cardiovascular system raises the intriguing possibility that histamine released by activated mast cells contributes to the observed cardiovascular dysfunction in EDS patients. Not only can histamine cause cardiovascular dysfunction, but in severe cases, its release can trigger the state of anaphylaxis [12].

Studies reveal elevated levels of established vascular inflammation markers in EDS patients. These markers include indicators of endothelial dysfunction (VCAM-1, ICAM-1, MCP-1) and an acute-phase reactant (CRP) [13]. A key question arising from this finding is whether these elevated markers translate into practical clinical applications for diagnosing or managing EDS. The elevated vascular inflammation markers observed in EDS patients might contribute to a cascade of events. These markers could potentially lead to endothelial dysfunction, the impairment of the blood vessel lining [13]. This endothelial dysfunction may then disrupt the proper functioning of smooth muscle cells, which could ultimately affect both the resting autonomic tone (baseline nervous system activity) and its reactivity (ability to respond to changes) [13].

We also found an increase in respiratory rate in patients with EDS which can be due to autonomic dysfunction. Costochondritis, bronchiectasis, and localized respiratory allergic and nonallergic mast cell activation are the most common inflammatory symptoms of tachypnea [14]. Breathing irregularities associated with autonomic dysregulation in the respiratory system include hyperventilation, Valsalva maneuvers, apnea, apneusis, breath holding, and rapid shallow breathing [15].

In light of the limitations of this study, we recommend conducting comprehensive longitudinal research with a larger number of samples in order to obtain more conclusive results. There were not many patients in the trial. Given that both genders were represented in the study sample, a further investigation emphasizing only women must be carried out. There have been contradictory findings involving autonomic reactivity in various EDS types; thus, further research is needed in this area.

## Conclusions

Our investigation suggests a potential link between EDS and autonomic nervous system dysfunction. The study identified signs of sympathoexcitation and reduced parasympathetic tone in EDS patients. These imbalances in the autonomic nervous system may contribute to the observed clinical features, including tachycardia (fast HR) and a drop in SBP (hypotension). Based on these findings, a potential link between EDS and autonomic nervous system dysfunction is suggested. This gold standard test establishes tools for assessing autonomic function. These tests could potentially serve a dual purpose: firstly, these tests may serve as a non-invasive screening method to identify the presence and severity of autonomic dysfunction in EDS and secondly, the results of these tests might offer valuable insights into the long-term risk to predict morbidity and mortality of cardiovascular complications in EDS patients.
